# *Prochlorococcus* extracellular vesicles: molecular composition and adsorption to diverse microbes

**DOI:** 10.1111/1462-2920.15834

**Published:** 2021-11-12

**Authors:** Steven J. Biller, Rachel A. Lundeen, Laura R. Hmelo, Kevin W. Becker, Aldo A. Arellano, Keven Dooley, Katherine R. Heal, Laura T. Carlson, Benjamin A. S. Van Mooy, Anitra E. Ingalls, Sallie W. Chisholm

**Affiliations:** ^1^ Department of Civil and Environmental Engineering Massachusetts Institute of Technology Cambridge MA USA; ^2^ Department of Biological Sciences Wellesley College Wellesley MA USA; ^3^ School of Oceanography University of Washington Seattle WA USA; ^4^ Department of Marine Chemistry and Geochemistry Woods Hole Oceanographic Institution Woods Hole MA USA; ^5^ Present address: GEOMAR Helmholtz Centre for Ocean Research Kiel Kiel Germany; ^6^ Present address: Integral Consulting Inc Seattle, WA USA; ^7^ Present address: Department of Biology Massachusetts Institute of Technology Cambridge MA USA

## Abstract

Extracellular vesicles are small (~50–200 nm diameter) membrane‐bound structures released by cells from all domains of life. While vesicles are abundant in the oceans, their functions, both for cells themselves and the emergent ecosystem, remain a mystery. To better characterize these particles – a prerequisite for determining function – we analysed the lipid, protein, and metabolite content of vesicles produced by the marine cyanobacterium *Prochlorococcus*. We show that *Prochlorococcus* exports a diverse array of cellular compounds into the surrounding seawater enclosed within discrete vesicles. Vesicles produced by two different strains contain some materials in common, but also display numerous strain‐specific differences, reflecting functional complexity within vesicle populations. The vesicles contain active enzymes, indicating that they can mediate extracellular biogeochemical reactions in the ocean. We further demonstrate that vesicles from *Prochlorococcus* and other bacteria associate with diverse microbes including the most abundant marine bacterium, *Pelagibacter*. Together, our data point toward hypotheses concerning the functional roles of vesicles in marine ecosystems including, but not limited to, possibly mediating energy and nutrient transfers, catalysing extracellular biochemical reactions, and mitigating toxicity of reactive oxygen species.

## Introduction

Many, if not all, bacteria release extracellular vesicles from their surface into the local environment (Deatherage and Cookson, 2012). In exponentially growing Gram‐negative bacteria, these structures are thought to derive primarily from the outer membrane, wherein a local region of membrane separates from the cell, carrying with it periplasmic material and other cellular components (Schwechheimer and Kuehn, 2015). A small subset of vesicles from Gram‐negative cells may include both outer and inner membrane material as well, further expanding the range of potential vesicle contents (Pérez‐Cruz *et al*., 2015). Vesicles are released constitutively during growth, but release rates can also vary in response to environmental perturbations (MacDonald and Kuehn, 2013; Biller *et al*., 2014). Extracellular vesicles represent a versatile secretion mechanism for cells (Schwechheimer and Kuehn, 2015; Guerrero‐Mandujano *et al*., 2017), and many classes of cellular compounds, including proteins, nucleic acids, and small molecules, have been identified within bacterial vesicles (Schwechheimer and Kuehn, 2015; Brown *et al*., 2015). Since they are bounded by a lipid bilayer membrane, vesicles also provide a mechanism for secreting and transporting hydrophobic compounds through aqueous extracellular environments (Mashburn‐Warren and Whiteley, 2006).

Extracellular vesicles can shuttle their contents between cells (Kadurugamuwa and Beveridge, 1996; Yaron *et al*., 2000). This ability enables vesicles to mediate a wide variety of biological functions such as horizontal gene transfer, signalling, pathogenesis, quorum signalling, biofilm development, nutrient exchange, viral interactions, and cellular defence (Kadurugamuwa and Beveridge, 1996; Yaron *et al*., 2000; MacDonald and Kuehn, 2012; Schwechheimer and Kuehn, 2015; Lynch and Alegado, 2017; Schatz *et al*., 2017). Although such exchanges have been shown to occur both among bacteria and across domains in a few laboratory models, the ‘rules’ dictating these exchanges are not at all clear, and essentially nothing is known about what occurs between cells and vesicles in complex microbial systems such as those found in the oceans, soils, or within the human microbiome.

Extracellular vesicles can reach concentrations of >10^5^ and >10^6^ ml^−1^ in open ocean and coastal waters, respectively (Biller *et al*., 2014) and represent an entirely new dimension of dissolved organic carbon pools. Their biological and ecological roles are unknown, but we do know that they are released by diverse taxa of both autotrophic and heterotrophic marine microbes, including the abundant cyanobacterium *Prochlorococcus* (Biller *et al*., 2014). With a global population of ~3 × 10^27^ cells, *Prochlorococcus* is an important primary producer, responsible for nearly 10% of marine net primary production (Flombaum *et al*., 2013). This group is known to secrete a number of organic compounds (Bertilsson *et al*., 2005) that provide marine heterotrophs with a source of carbon and energy (Ottesen *et al*., 2014; Becker *et al*., 2019) and contribute to marine dissolved organic carbon pools. *Prochlorococcus* extracellular vesicles represent at least one component of this labile organic photosynthate, as purified vesicles have been shown to support the growth of a marine heterotroph (Biller *et al*., 2014).

Here, we explore the contents and function of extracellular vesicles produced by *Prochlorococcus*, focusing on their potential contribution to ocean dissolved organic matter and their ability to interact with other marine microbes. We characterized the lipidome, proteome, and metabolome of vesicles released by two ecologically distinct strains and use these inventories to develop hypotheses of vesicle functions. To establish whether *Prochlorococcus* vesicles can chemically interact with surrounding seawater, we determined whether they contain active enzymes. Finally, we investigated the potential for *Prochlorococcus* vesicles to mediate biotic interactions by studying whether they can form specific associations with other strains representative of abundant marine microbes.

## Results and discussion

### Diverse biomolecules are associated with *Prochlorococcus* extracellular vesicles

We used a combination of targeted and untargeted ‘omics’ approaches to examine the contents of vesicles and the cells that released them. Our study focused on vesicles isolated from exponentially growing, asynchronous cultures of two ecologically distinct *Prochlorococcus* – MIT9312, a high‐light adapted strain, and MIT9313, a low‐light adapted strain (Biller *et al*., 2015a,b). We uncovered a vast diversity of biomolecules in these discrete, lipid‐bound, colloidal packets, as detailed below.

#### Lipids and pigments

**Fig. 1:**
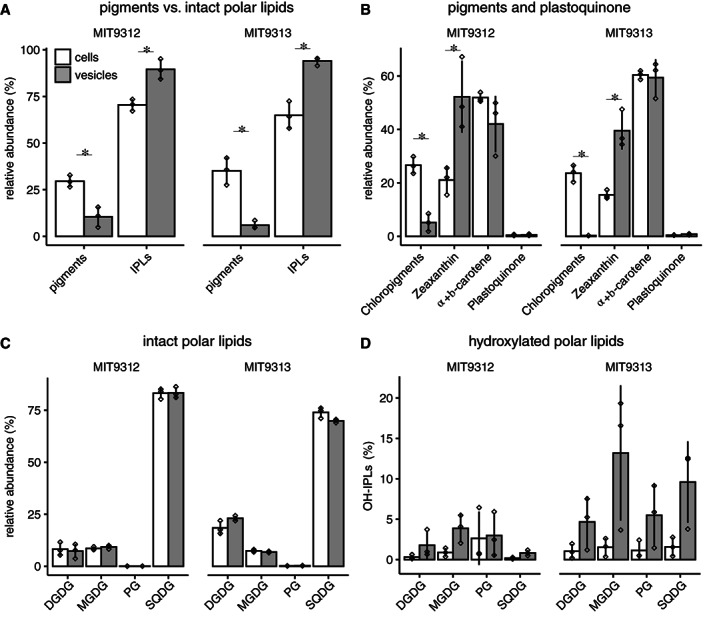
Lipid and pigment content of *Prochlorococcus* cells and vesicles from two strains, MIT9312 and MIT9313. A. Relative abundance of pigments vs intact polar lipids (IPLs) in vesicles and cells. B. Relative abundance of specific pigments and plastoquinone. C. Relative abundance of different IPL groups. D. Fraction of hydroxylated polar lipids (OH‐IPLs) within each IPL class. Values indicate the mean (±SD) of three biological replicates of strains MIT9312 (white) and MIT9313 (black). MGDG: monoglycosyl diacylglycerol; DGDG: diglycosyl diacylglycerol; PG: phosphatidylglycerol; SQDG: sulfoquinovosyl diacylglycerol. * indicates significant differences between cells and vesicles (two‐tailed *t* test, *P* < 0.05).

Lipids from vesicles and cells of both *Prochlorococcus* strains were analysed using an untargeted, high‐resolution mass spectrometry‐based approach. Vesicles contained a suite of different intact polar lipids (IPLs), various pigments, and plastoquinone. Overall, vesicles were relatively enriched for IPLs verus pigments, as compared with the parent cells (Fig. 1A; Supporting Information Table S1). The carotenoids zeaxanthin and carotene were the most abundant pigments in vesicles from both strains, which contained relatively few chloropigments such as divinyl chlorophylls *a* and *b* (Fig. 1B; Supporting Information Table S1). While plastoquinone was identified in the vesicles (Fig. 1B; Supporting Information Table S1), they contained little thylakoid material overall, suggesting that vesicle carotenoids could come from other cellular locations such as the outer membrane, as has been shown for *Synechocystis* PCC6714 (Jürgens and Weckesser, 1985).

*Prochlorococcus* cellular membranes are primarily composed of sulfolipids, reflecting an adaptation to the low phosphorous oligotrophic environment in which they live (Van Mooy *et al*., 2006). This was evident in the lipid composition of its extracellular vesicles as well. The IPL composition of *Prochlorococcus* extracellular vesicles was dominated by sulfoquinovosyl diacylglycerol (SQDG), diglycosyl diacylglycerol (DGDG) and monoglycosyl diacylglycerol (MGDG) – each found in similar relative abundances (as a proportion of all IPLs) in cells and vesicles (Fig. 1C; Supporting Information Table S1). The overall vesicle lipid composition of each strain more closely resembled the composition of the parent cells than the vesicles from the other strain (Figs. 1C and S1), consistent with our previous observations from *Prochlorococcus* strains MIT9313 and MED4 (Biller *et al*., 2014) suggesting that there is not a universal *Prochlorococcus* vesicle lipidome. Vesicles from the two strains did, however, share some common lipid features that distinguished them from the parental cells. Of note, hydroxylated forms of all major IPLs identified (polar lipids modified by oxidation reactions) were relatively more abundant in vesicle samples as compared with cells (Fig. 1D; Supporting Information Table S1). In addition, the fraction of some hydroxylated IPLs (DGDG in MIT9312; SQDG and MGDG in MIT9313) among biological replicate vesicle samples was significantly more variable than in the corresponding cellular samples (*F*‐test, *P* < 0.05; Figs. 1D and S1B–D). Annotation of these hydroxylated lipids revealed that the hydroxylation is site specific, for example, at the Δ9‐position of a C_18:2_ fatty acid (Supporting Information Fig. S2) regardless of head group. The exact oxidative mechanism (enzymatic, radical mediated, or non‐radical mediated) responsible for generating the hydroxylation is not known, but free‐radical (auto) oxidation at a specific double bond would be expected to result in at least four isomers of similar abundance (Rontani and Belt, 2020). While lipid hydroxylation could have occurred either before or after vesicle release, the site‐specificity raises the possibility that they could be of biological origin. One potential explanation is that *Prochlorococcus* outer membranes are generally enriched in hydroxylated lipids relative to other membranes, as has been shown for other Gram‐negative bacteria (Schmidt *et al*., 1980; Volkman *et al*., 1998). This would be consistent with their enrichment in outer membrane‐derived vesicles and perhaps reflect a role for vesicle secretion as a mechanism for removing damaged lipids from the cell. Hydroxylipids may also be involved in the membrane curvature and bending processes required for vesicle formation, leading to their preferential incorporation and export.

#### Proteins

Since many of the functional roles attributed to vesicles in other microbial systems are due to the activity of proteins (Schwechheimer and Kuehn, 2015), we explored the global proteomes of the vesicle and cellular fractions of two *Prochlorococcus* strains using a label‐free, quantitative shotgun proteomics approach. During extraction and sample preparation, we utilized surfactants compatible with mass spectrometry to improve the recovery of more membrane‐bound proteins and better facilitate in‐solution protease digestion, which further helped generate more comprehensive vesicle and cellular proteomes. MIT9312 and MIT9313 vesicles contained, respectively, at least 11% and 12.5% of all predicted proteins encoded by the cell's genome (Table 1). This most likely does not represent every protein found in vesicles, but rather what could be detected within the relatively small amount of vesicle biomass we could obtain from 20 l cultures as compared with cellular material (c.f., cellular proteomes for MIT9312 and MIT9313 recovered nearly 52% and 37% of all predicted proteins, respectively; Table 1). That said, these vesicle proteomes yielded notably more protein identifications than our previous study of MIT9313 and MED4 vesicles (in which gel‐extracted protein bands were analysed; Biller *et al*., 2014), and it is noteworthy that 25 of the 27 proteins we previously identified in MIT9313 were also present in this data set. Further, those 25 were among the top 50 most highly abundant proteins in the MIT9313 vesicle proteome (Supporting Information Tables S2 and S3). The concordance of these results shows that *Prochlorococcus* cells grown under similar conditions will reproducibly package and export intact proteins within vesicles.

**Table 1:** Number of proteins identified in *Prochlorococcus* cells and vesicles.

Strain	Annotated proteins encoded in genomes (Uniprot)	Annotated proteins shared by both genomes	Proteins detected in cells^a^	Proteins detected in cells of both strains	Proteins detected in vesicles	Proteins detected only in vesicles and not in cells	Proteins detected in vesicles from both strains
MIT9312	1964	1532	1030	714	224	17	111
MIT9313	2830		1055		355	41	

^a^ These values reflect the data gathered for the trypsin/Lys‐C digestion of *Prochlorococcus* cells and vesicles (Supporting Information Tables S2 and S3); not included here, additional cellular proteins were identified using Glu‐C digestion.

**Fig. 2:**
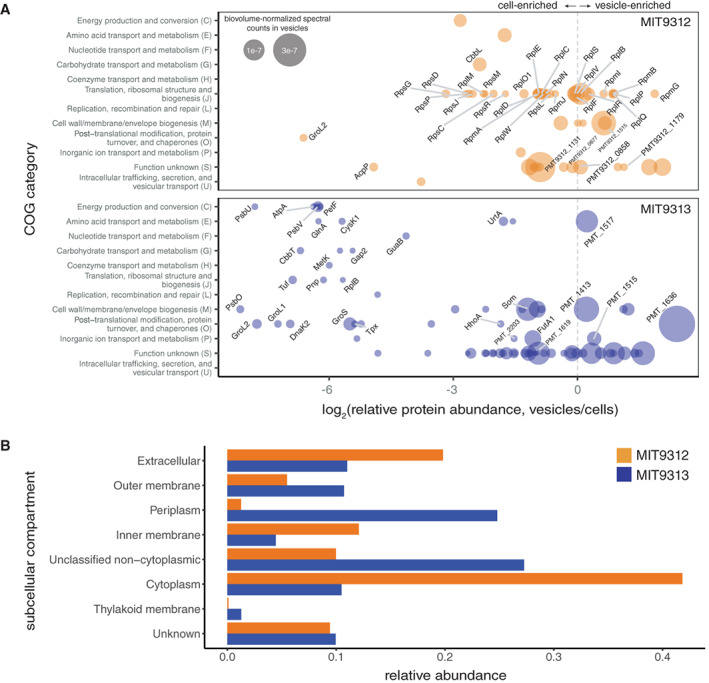
*Prochlorococcus* vesicle proteomes. A. Relative protein enrichment in *Prochlorococcus* vesicles compared with cells. Points represent the log_2_ ratio of relative protein abundance for the top 25% most abundant proteins identified in vesicles of strain MIT9312 (above; orange) or MIT9313 (below; blue), as grouped by NCBI clusters of orthologous groups of proteins (COG) functional categories. The area of each point indicates biovolume‐normalized spectral counts (abundance) of that protein within the vesicle proteome. Names or locus tags are indicated for selected proteins; data for all vesicle proteins are found in the Supporting Information Table S4. B. Relative abundance of proteins found in MIT9312 (orange) and MIT9313 (blue) vesicles, as grouped by predicted subcellular localization (see Methods).

Vesicles produced by the two *Prochlorococcus* strains contained some proteins in common (Tables 1 and S2), but we also noted strain‐specific differences, as has been found in comparisons of vesicles from other closely related microbes (Tandberg *et al*., 2016; Bitto *et al*., 2021). The overall *Prochlorococcus* vesicle proteome included proteins associated with many functional categories (Figs. 2A, S3). For instance, both MIT9312 and MIT9313 vesicles contained putative porins and transporters (e.g. PMT9312_1131, Som), peptidases/hydrolases (e.g. PMT9312_0677, PMT_1636), chaperones (e.g. DnaK and DnaJ), and uncharacterized proteins (Fig. S3; Table S4). The relative abundance of these shared proteins did, however, vary between strains (Fig. 2A; Tables [Link], [Link]). Proteins uniquely found in MIT9312 vesicles included a cAMP phosphodiesterase (PMT9312_0858), an adhesin‐like protein (PMT9312_1179), a phosphate ABC transporter substrate‐binding protein (PstB), and some ribosomal proteins. Among those found uniquely in MIT9313 were multiple ABC transporter binding proteins (e.g. UrtA, FutA1, PMT_2203), a sulfatase (PMT_1515), and a putative phosphatase (PMT_1619) (Fig. 2A; Tables S2 and S3). Thus, the vesicles released by two relatively similar organisms have distinct functional potentials.

The functional potential of vesicles will also be influenced by the relative abundance of any individual protein within the vesicle population. Indeed, different proteins were packaged at different levels within the vesicles in our samples, with a subset enriched (on a biovolume‐normalized basis) in vesicles relative to cells (Figs. 2A, S4; Table S4). To explore what factors might influence the packaging, we examined whether subcellular localization correlated with vesicle export. Consistent with a primarily outer membrane origin for extracellular vesicles, most outer membrane and periplasmic proteins detected in the cellular proteome were identified at some level within vesicles (Fig. S5). As seen in other Gram‐negative bacteria (Pérez‐Cruz *et al*., 2015; Zakharzhevskaya *et al*., 2017; Yun *et al*., 2017), we found proteins from all major cellular regions to be exported within *Prochlorococcus* vesicles (Fig. 2B). We were, however, surprised to find that cytoplasmic proteins made up the largest fraction in strain MIT9312, whereas most proteins in MIT9313 vesicles were predicted to originate from various locations outside the cytoplasm (Fig. 2B). Although a number of abundant cytoplasmic proteins, such as ribosomal proteins, were found in MIT9312 vesicles (Fig. 2A; Tables S2 and S3), there was no general relationship between their abundance in the cells and vesicles (Supporting Information Fig. S6A). Together with the relative lack of thylakoid proteins and chlorophyll in the vesicles, this argues that the presence of cytosolic proteins is likely not due to artefacts such as cell lysis. We also saw no clear relationship between a protein's presence in the vesicles and properties such as isoelectric point or molecular weight (Supporting Information Fig. S6B–E).

A number of factors likely contribute to the differences observed between the vesicle proteomes of the two strains. Some differences are simply attributable to strain‐specific genomic differences (Table 1). While the two genomes share over 1500 genes, MIT9313 encodes ~870 more genes than does MIT9312, and its vesicles contained a proportionally more diverse set of proteins. One notable example was the identification of several prochlorosins, or ProcA peptides, within the vesicle proteome of MIT9313 (Supporting Information Table S2). Prochlorosins are cyclic peptide secondary metabolites encoded by some low‐light adapted, but not high‐light adapted, *Prochlorococcus* – including 29 diverse *procA* genes in MIT9313 (Li *et al*., 2010; Cubillos‐Ruiz *et al*., 2017). Strain‐specific genome differences do not, however, explain all of the vesicle proteome variation. Even when considering only the proteins shared by both strains, we found that the relative abundance of orthologous proteins was linearly correlated in the whole cell fraction, but that there was no clear relationship in the relative amounts of these proteins within extracellular vesicles (Supporting Information Fig. S4B). We speculate that differences in cellular structure between the strains (Ting *et al*., 2007) may have influenced the differential incorporation of proteins from the cytosol and other subcellular compartments into vesicles.

While we cannot rule out the possibility that some individual proteins were specifically packaged via unknown mechanisms, our data suggest that most proteins are packaged stochastically, with the probability of export influenced by a number of factors including initial cellular protein abundance, subcellular localization, and perhaps strain‐specific differences in cellular architecture. Physical constraints, such as the amount of membrane relative to the aqueous lumen of the vesicle, may also influence protein composition and limit the enrichment of some compounds in vesicles relative to cells (Turner *et al*., 2018). Finally, it is likely that additional sampling would reveal an even longer ‘tail’ of other proteins that are either exported at relatively lower abundances or were perhaps lost through the purification protocols used here. Thus, we suspect that most (or perhaps all) proteins expressed within cells will eventually be found within vesicle populations. Future work examining vesicles across strains (Tartaglia *et al*., 2020; Zwarycz *et al*., 2020) and under different growth conditions (Orench‐Rivera and Kuehn, 2016; Zavan *et al*., 2019) will be needed to advance our understanding of the mechanisms influencing vesicle proteome composition, and how such differences might affect the emergent functional impact of those vesicles on the ecosystem.

#### Metabolites

A combination of targeted and untargeted metabolomics analysis identified 1662 and 2035 unique mass features in vesicle populations from *Prochlorococcus* MIT9312 and MIT9313, respectively (Supporting Information Table S5). These mass features represented molecules across a range of *m/z* values (~94 to ~800), polarities, and charge states (Supporting Information Table S6). Most vesicle‐associated metabolites detected were non‐polar (90% and 85% in MIT9312 and MIT9313 vesicles, respectively), whereas non‐polar metabolites represented 52%–56% of mass features detected in the cells (Tables S5 and S6). The identification of diverse non‐polar molecules in vesicles further supports the idea that extracellular vesicles can serve as vehicles for the secretion of hydrophobic compounds (Mashburn and Whiteley, 2005; Schertzer *et al*., 2009), facilitating biological activities that depend on extracellular transport of nonpolar molecules within aqueous environments.

While we are not able to identify most of the observed mass features, we could document a number of known compounds within the vesicles (Table 2). For instance, vesicles from both strains contained phylloquinone (Vitamin K1), a compound involved in electron transfer. We also identified a number of oxidized carotenoid products (Table 2) in vesicles from both strains, though it is unclear whether these carotenoids were oxidized in the cell and then exported via vesicles or whether they became oxidized following vesicle release. Although we focused our analysis on the compounds present in vesicles, cellular compounds absent in vesicles are also of interest (Table S7). For example, *Prochlorococcus* cells contained potential osmolytes such as sucrose, glycine betaine, or glucosylglycerol, which help maintain cellular osmotic balance in cyanobacteria (Klähn and Hagemann, 2010), but these compounds were undetectable in either set of vesicles (Supporting Information Table S7). This represents a puzzle as to how cytoplasmic material could be exported to vesicles without including (or retaining) the most abundant solutes in the cell and raises questions concerning how vesicles may change in response to osmotic stresses.

**Table 2:** Non‐intact polar lipid metabolites identified within *Prochlorococcus* vesicles.

Compound name	Fraction	Compound class	MIT9312 vesicles	MIT9313 vesicles	Confidence level	*m/z*	RT (min)
Triose	HILICAqNeg	Sugar	+	+	3a	503.1629	13.6
Tetraose	HILICAqNeg	Sugar	+	+	3a	665.2164	14.4
Phylloquinone (Vitamin K1)	RPOrgPos	Electron transfer	+	+	1	451.3572	14.6
Carotene	RPOrgPos	Pigment	+	+	1	536.4371	16.1
Protoporphyrin	RPOrgPos	Pigment or cyt‐c precursor	+	+	2	563.2656	12.8
1‐Lauroyl‐glycerol (C12)	RPOrgPos	Lipid	+	+	2	257.2111	11.7
1‐Myristoyl‐glycerol (C14)	RPOrgPos	Lipid	+	+	2	285.2423	13.2
Beta‐Apo‐12'‐carotenal	RPOrgPos	Pigment oxidation product	+	+	3b	351.2682	13.5
Apo‐12'‐zeaxanthinal	RPOrgPos	Pigment oxidation product	+	+	3b	367.2633	12.0
Beta‐Apo‐10'‐carotenal	RPOrgPos	Pigment oxidation product	+	+	3b	377.2839	13.8
Apo‐10'‐zeaxanthinal	RPOrgPos	Pigment oxidation product	+	+	3b	393.2785	12.4

For details on analytical fractions and confidence level assessments, see Methods and Supplementary Information. + = detected.

### Nutrient and energy sources within vesicles

*Prochlorococcus* vesicles have been shown to serve as sources of organic carbon and/or energy for supporting growth of co‐cultured bacteria (Biller *et al*., 2014). Much of a vesicle's carbon and chemical energy content is found within the lipids, as each 100 nm diameter vesicle will contain ~10 000 lipid molecules in its envelope. The diverse proteins and metabolites exported within vesicles represent potential labile organic carbon sources as well. In one notable example, vesicles from both strains contained triose and tetrose sugars (Table 2), which were not measurable above background levels within cells. While not the focus of this work, *Prochlorococcus* vesicles contain DNA and RNA (Biller *et al*., 2014) as well, and vesicle‐associated nucleic acids may also function as an energy or nutrient source for microbes (Jørgensen and Jacobsen, 1996).

We previously observed nutrient binding proteins, such as those for urea, phosphate, and iron, in the vesicles exported by *Prochlorococcus* strains MED4 and MIT9313 (Biller *et al*., 2014). This suggested that vesicles might transport nutrients or even scavenge compounds as they diffuse through seawater and, in turn, organisms that encounter these vesicles could gain access to a locally concentrated ‘packet’ of nutrients. To continue to explore this hypothesis, we looked for annotated substrate‐binding proteins and transporters in the proteome data. We again identified putative substrate‐binding proteins (many associated with ABC transporters) for urea, phosphate, and iron in the vesicles, along with those for manganese and amino acids (Supporting Information Tables S2 and S3). Vesicles produced by both strains contained putative transporters for sulfate, magnesium, and ammonium (Supporting Information Tables S2 and S3), raising the possibility that – assuming favourable energetics and proper protein orientation in the membrane – vesicles could accumulate these compounds following cellular release.

As a first step toward determining whether the nutrients that the proteomic data suggest might be transported by vesicles are actually present, we measured vesicle phosphate content and compared it with that of the average cell. Vesicles from MIT9312 and MIT9313 contained 3.4 ± 3.2 and 3.7 ± 2.1 femtomoles phosphate per 10^6^ vesicles, respectively. Given that a *Prochlorococcus* MED4 cell (similar in size to MIT9312) contains ~10 attomoles phosphorus (P) (Bertilsson *et al*., 2003) and *Pelagibacter* HTCC7211 cells contain ~16 attomoles P (White *et al*., 2019) under P‐limited conditions, an encounter with a single *Prochlorococcus* vesicle would supply only ~0.01% of these cells' P quota. These are averages, however, and absent an understanding of the distribution of P per vesicle and vesicle‐cell encounter rates it is impossible to speculate further; we simply flag this as an interesting question to pursue. We also note that the presence of nucleic acids in vesicles represents a possible source of organic phosphorous.

As has been observed in gram‐negative pathogens (Pérez‐Cruz *et al*., 2015), vesicles from both *Prochlorococcus* strains contained ATP, with more released by MIT9312 than MIT9313 (c.f., 3.6 ± 1.4 × 10^−3^ and 0.13 ± 0.097 × 10^−3^ femtomoles ATP per 10^6^ vesicles, respectively). This has relevance for interpreting measurements of ‘dissolved’ ATP in the <0.2 μm fraction of seawater (Azam and Hodson, 1977) – which we now know contains vesicles. This dissolved ATP can be utilized by marine microbes (Azam and Hodson, 1977) and has been proposed to arise largely from cellular excretion during active growth and/or grazing activity, as opposed to lysis of dead cells (Nawrocki and Karl, 1989; Björkman and Karl, 2001). Our results suggest that at least some fraction of this dissolved ATP excreted by marine microbes is likely associated with extracellular vesicles.

### *Prochlorococcus* vesicles contain catalytically active enzymes

**Fig. 3:**
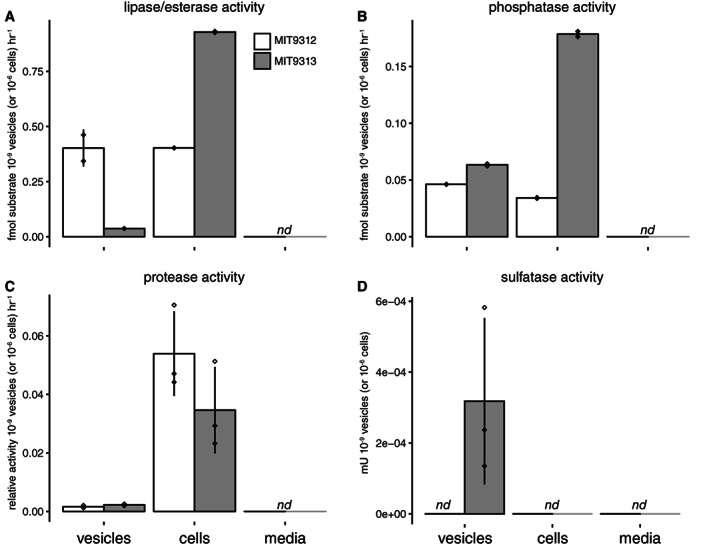
Enzymatic activity in purified *Prochlorococcus* vesicles. Activity of (A) lipases, (B) phosphatases, (C) proteases, and (D) sulfatases in samples of vesicles from *Prochlorococcus* MIT9312 (white) and MIT9313 (grey) are compared to that of cells and media background. Values represent the mean (±SD) of two to three biological replicates, standardized per population of either 10^9^ vesicles or 10^6^ cells. *nd* = not detected.

Many of the most abundant proteins in the vesicles were putative enzymes (Supporting Information Table S2) which have the potential to mediate exoenzymatic activities. We conducted a set of *in vitro* biochemical assays with intact, purified vesicles and showed that they exhibit lipase, phosphatase, protease, and sulfatase activity (Fig. 3). Vesicle‐associated enzymes are thus able to access substrates in the media, likely either via leakage of compounds across the membrane, transport into the vesicle interior, or by acting directly on substrates outside of the vesicle. We also observed that substrate accessibility can differ between vesicles and cells: *Prochlorococcus* MIT9313 vesicles displayed measurable sulfatase activity, while intact cells did not (Fig. 3D). Thus, vesicle‐associated enzymes have the potential to act upon extracellular substrates that they might not encounter within the cell (Ebner and Götz, 2019), suggesting that many proteins typically thought to be cytosolic could contribute to extracellular biogeochemical processes *in situ*.

We speculate that exporting enzymes in vesicles could be a means of protecting the proteins from environmental damage and degradation, allow for simultaneous co‐secretion of multiple proteins, and/or provide an environment where substrates could be maintained in close proximity in the otherwise dilute oligotrophic ocean (Bonnington and Kuehn, 2014). Given that osmolytes can play important roles in maintaining enzyme activity within cells (Yancey, 2005), it is also intriguing that these vesicle‐associated enzyme activities are maintained in the absence of the most abundant detectable cellular osmolytes. While cyanobacteria and many other microbes specifically secrete certain exoenzymes into their local environment (Christie‐Oleza *et al*., 2015), the broad diversity of the vesicle proteome seen here indicates that most cellular enzymes may be exported at some level within vesicles and thus have the potential to function extracellularly.

### Can marine extracellular vesicles interact with cells from different organisms?

**Fig. 4:**
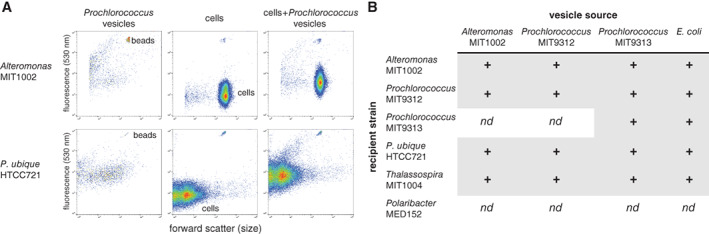
Association of extracellular vesicles with diverse microbial cells. A. Example flow cytometry plots of fluorescently labelled *Prochlorococcus* MIT9313 vesicles (left), *Alteromonas* MIT1002 and *Pelagibacter ubique* (SAR11) cell populations (centre), and the same cells following a 2 h incubation with vesicles (right). Internal reference beads are labelled. B. Interaction of labelled vesicles with cells (+), or lack thereof (*nd* = not detected), for different ‘source’ strains (vesicle producers) and ‘recipient’ strains (cells exposed to labelled vesicles). Positive interactions were determined based on two criteria: an increase in normalized median cellular 530 nm fluorescence when mixed with labelled vesicles (as in a; see also Fig. S7), and a statistically significant shift in the cellular population distribution (χ² T(x) test, *P* << 0.01 in two to three biological replicates; see Supplementary Methods). *Alteromonas* and *Prochlorococcus* vesicles were covalently labelled with an amine‐reactive Alexa 488 dye; *E. coli* vesicles contained GFP.

We know from studies of terrestrial and host‐associated microbes that many biological functions of vesicles are attributable to the transfer of vesicle contents between cells (Schwechheimer and Kuehn, 2015; Brown *et al*., 2015). Vesicle‐mediated biotic ‘interactions’ may encompass a range of mechanisms, from vesicle‐associated activities occurring in proximity to a cell (e.g. enzymatic activities or phage defence; MacDonald and Kuehn, 2012; Biller *et al*., 2014; Rakoff‐Nahoum *et al*., 2014) to the delivery of vesicle contents into another cell (Kadurugamuwa and Beveridge, 1999). To begin to explore interactions between *Prochlorococcus* vesicles and cells, we looked for physical associations between fluorescently labelled vesicles and six different marine microbes from the Proteobacteria, Cyanobacteria, and Bacteroidetes – the three most abundant bacterial phyla in the oceans (Fig. 4). We found that vesicles from *Prochlorococcus* MIT9313 associated with cells of both *Prochlorococcus* MIT9312 and MIT9313, along with representatives of the marine Gammaproteobacteria and Alphaproteobacteria – including *Candidatus Pelagibacter ubique* HTCC7211, a member of the numerically dominant SAR11 group of marine heterotrophs (Figs. 4 and S7). The breadth of interactions was not specific to *Prochlorococcus* vesicles, as vesicles purified from the marine heterotroph *Alteromonas* MIT1002, which was co‐isolated with *Prochlorococcus* (Biller *et al*., 2015b), were also able to interact with these strains (Figs. 4 and S7). To ensure that the observed vesicle‐cell associations were not an artefact of the fluorescent dye used, we incubated the same set of marine microbes with vesicles from an *E. coli* strain (Dinh and Bernhardt, 2011) which instead expressed GFP within vesicles, and found that they were also able to interact with multiple microbes (Figs. 4B and S7). While we further verified that our fluorescent vesicle labelling approach did not significantly influence the ability of *E. coli* vesicles to interact with cells (Fig. S8), we cannot rule out a potential influence of vesicle surface modifications on the pairwise interactions tested.

Four of the strains examined interacted with all of the vesicles tested (Fig. 4B) while two others exhibited apparent specificity toward vesicles from different sources. That is, vesicles from both *Alteromonas* and *Prochlorococcus* MIT9312 did not associate with *Prochlorococcus* MIT9313 cells, and none of the vesicles interacted with *Polaribacter* MED152, a marine *Bacteroidetes* (González *et al*., 2008), at our detection levels (Fig. 4B). This confirms that our observations are not simply due to experimental conditions or random ‘sticking’ of vesicles to cells. Specificity in vesicle‐cell associations has been shown in some heterotrophic bacteria (Tashiro *et al*., 2017; Toyofuku *et al*., 2017) and likely underlies variation in vesicle‐mediated DNA transfer rates among species (Tran and Boedicker, 2017). Our data are consistent with the hypothesis that different vesicle‐cell pairs may vary in the strength of their interactions (Supporting Information Fig. S7). For example, the propensity of a vesicle to associate with a particular cell may depend on its specific surface composition, which would likely be obscured by the averaging effect of our population‐scale analysis. The apparent lack of vesicle associations with *Polaribacter* in our experiments is striking and invites an exploration of what factors, such as surface charge (Tashiro *et al*., 2017), cell envelope structure, and surface hydrophobicity (MacDonald and Beveridge, 2002), might define interaction boundaries. More generally, these results raise new questions concerning the degree to which vesicles should be considered ‘public goods’ in the oceans.

The vesicle‐cell associations could be disrupted by repeated rounds of centrifugation and washing of the cells (Supporting Information Fig. S9), suggesting that the majority of labelled vesicle material was not fully integrated into the outer membrane of the cells (at least at this timepoint) but was attached via non‐covalent bonding between the two surfaces. Although these observations do not directly establish a particular biologically relevant function, we speculate that ‘captured’ vesicles could serve many functions, including delivering cargo through membrane fusion, ‘flipping’ of molecules from the vesicle membrane into the cell (Remis *et al*., 2014), or extracellular degradation and subsequent uptake of vesicle contents. Alternatively, the presence of vesicles nearby could serve defensive roles for cells (Manning and Kuehn, 2011) or influence cellular processes via enzymatic activities.

### Potential functional roles for *Prochlorococcus* vesicles in marine ecosystems

The diversity of extracellular vesicle contents observed here is consistent with the idea that vesicles do not have one dedicated function but instead may play a variety of roles within microbial ecosystems. Our detailed inventory of *Prochlorococcus* extracellular vesicle contents indicates potential functional impacts of vesicles specific to dilute ocean ecosystems that merit future exploration from both conceptual and quantitative perspectives. There are a broad range of compounds within vesicles that could be exchanged between microbes or serve other roles in these particular ecosystems. For instance, the triose and tetrose sugars could serve as an additional energy or carbon source for organisms, act as antioxidants, or possibly serve as the primary osmolyte within vesicles (Goh *et al*., 2010). While there may be only ~2 molecules of ATP within any individual vesicle on a bulk‐average basis, the aggregate impact of vesicle‐associated ATP exchange among cells remains to be established; further, the presence of proteins and ATP within the same vesicle could facilitate ATP‐requiring extracellular reactions or enable other types of roles such as signalling. Many additional possible ecological functions for vesicles are likely reflected in the other proteins and metabolites found in our inventory of *Prochlorococcus* vesicles, which we have not specifically discussed here.

The identification of diverse oxidized compounds in *Prochlorococcus* vesicles (Table 2 and Fig. 1D) raises additional questions concerning the role vesicles may play in dissipating oxidative stress – a particular challenge for *Prochlorococcus*, which lacks enzymes typically used for detoxification of hydrogen peroxide such as catalase (Scanlan *et al*., 2009). Vesicles could be acting as a mechanism for removing damaged compounds, complementing other cellular metabolite degradation and repair pathways (Linster *et al*., 2013), or as a ‘sink’ for reactive oxygen species (ROS) generated within the cell, within vesicles, or in the extracellular seawater environment (Morris *et al*., 2016). Regardless of the oxidative mechanism(s) involved, suppression of ROS toxicity and/or removal of oxidized metabolites may be another way in which vesicles serve a protective role for cells (Manning and Kuehn, 2011). Our identification of vesicle‐associated carotenoids, which have also been observed in vesicles from *Synechocystis* PCC6803 (Pardo *et al*., 2015), raise broader questions as to whether these compounds might serve a photoprotective role for vesicle contents.

Given the number of proteins and small molecules identified in the vesicles, it seems implausible that these materials are uniformly distributed among each member of the vesicle population. We have previously shown heterogeneity of DNA fragment incorporation into vesicles from multiple marine bacteria (Biller *et al*., 2017), and vesicle subpopulations containing both outer and inner membrane material from Gram‐negative cells have been reported (Pérez‐Cruz *et al*., 2015). Furthermore, distinct vesicle subpopulations with unique origins and epitopes have been noted for some time in eukaryotic cells (Kowal *et al*., 2016). Thus, the functional potential of one vesicle is likely different from the next – similar to our perspective on the cells from which vesicles are derived, whose ecological role can only be understood by viewing them as populations of diverse entities, which may display a diverse range of functions in different contexts (Biller *et al*., 2015a).

### Conclusions

The abundance of vesicles in seawater, and the diversity of their chemical contents, prompt a reassessment of how we conceptualize and interpret the pool of dissolved organic matter that is so critical to the function of marine ecosystems. While many cellular compounds are secreted in the form of truly ‘dissolved’ individual molecules, it is now clear that microbes also release organic molecules in locally structured, colloidal vesicles which may influence the accessibility, activity, and half‐lives of their contents. As such, vesicles blur the boundaries between definitions of ‘particulate’ and ‘dissolved’, or ‘living’ and ‘detrital’, in marine biogeochemistry – distinctions that are critical for understanding and modelling the system. Extracellular vesicles represent a new frontier for understanding the flow of energy, materials, and information in ocean ecosystems.

## Experimental procedures

### Culturing conditions and sampling for lipidomics, proteomics, and metabolomics

To minimize media background signals, axenic cultures of *Prochlorococcus* MIT9312 and MIT9313 used for lipidomics, proteomics, and metabolomics analyses were grown in chemically defined artificial AMP1 media (Moore *et al*., 2007) supplemented with 10 mM (final concentration) filter‐sterilized sodium bicarbonate. All 20 l cultures were grown in polycarbonate carboys (ThermoFisher Nalgene, Waltham, MA, USA) with gentle stirring (60 rpm), under constant light flux (10–20 μmol Q m^−2^ s^−1^ for MIT9313; 30–40 μmol Q m^−2^ s^−1^ for MIT9312) at 24°C.

All cell and vesicle samples were collected during mid‐to‐late exponential growth phase. Cell pellets were obtained by gently centrifuging cells (7500 × *g*) for 10 min at 4°C; vesicle isolation is detailed below. For lipidomics, proteomics, and metabolomics analyses, a total of seven 20 l cultures were grown in AMP1 media for each of the two *Prochlorococcus* strains, providing three replicates for the lipid and small metabolite analysis and an additional sample for proteomics analysis. *Prochlorococcus* AMP1 growth media blanks and vesicle suspension buffer [phosphate buffered saline (PBS)] blanks served as controls for lipidomics, proteomics, and metabolomics analysis; they were extracted and analysed alongside all cell and vesicle samples. Since *Prochlorococcus* was grown under continuous light, the cultures were not synchronized. Thus, all cell samples represent a bulk average population of cells at all stages of the cell cycle; vesicle samples similarly integrate material released across both the lag and exponential growth (steady state) phases.

### Vesicle isolation

Vesicles were isolated as described previously (Biller *et al*., 2014). Briefly, cultures were first gravity filtered through a 0.2 μm capsule filter (Polycap 150TC; GE Life Sciences/Whatman, Maidstone, UK). The filtrate was then concentrated using a 100 kDa tangential flow filter (Ultrasette with Omega membrane; Pall, Port Washington, NY, USA) and re‐filtered through a 0.2 μm syringe filter. Vesicles were pelleted from the sample by ultracentrifugation at ~100 000 × *g* (Beckman‐coulter SW32Ti rotor; 32 000 rpm, 1.5 h, 4°C), purified on an OptiPrep gradient (Biller *et al*., 2014), then washed and resuspended in 0.2 μm filtered 1× PBS.

Vesicle concentrations were measured with a NanoSight LM10HS instrument equipped with a LM14 blue laser module using NTA software V3.1 (NanoSight/Malvern, Westborough, MA, USA). Samples were diluted such that the average number of particles per field was between 20 and 60, per the manufacturer's guidelines. Three replicate videos were collected from each sample at a camera level of 11 and analysed at a detection threshold of 1. The sample chamber was thoroughly flushed with 18.2 MΩ cm^−1^ water (Milli‐Q; Millipore, Burlington, MA, USA) between samples and visually examined to ensure that no particles were carried over.

### Lipidomics

Lipids were extracted from triplicate cell pellets and vesicles from *Prochlorococcus* MIT9312 or MIT9313 using a modified Bligh and Dyer protocol using a 2:1:0.8 ratio of methanol:dichloromethane:phosphate buffered saline (Popendorf *et al*., 2013). The total lipid extract was analysed on an Agilent 1200 reversed phase high‐performance liquid chromatograph (HPLC) coupled to a ThermoFisher Exactive Plus Orbitrap high‐resolution mass spectrometer (HRMS; ThermoFisher, Waltham, MA, USA) which scanned across a range of 100–1500 *m/z*. HPLC and MS conditions are based on methods described by Collins *et al*., 2016 (modified after Hummel *et al*., 2011) and are detailed in the Supporting Information. For the identification and peak area integration, we used LOBSTAHS, an open‐source lipidomics software workflow based on adduct ion abundances and several other orthogonal criteria (Collins *et al*., 2016). Lipid peaks identified using the LOBSTAHS software were integrated from MS data after pre‐processing with XCMS (Smith *et al*., 2006) and CAMERA (Kuhl *et al*., 2012) and corrected for differences in response factors among different lipids using commercially available standards as described by Becker *et al*. (2018). Additional details are available in the Supplementary Information.

### Proteomics

Proteins were extracted from cell biomass and vesicles from a single 20 l batch culture of *Prochlorococcus* MIT9313 or MIT9312. Filters containing cell biomass or vesicle suspensions were extracted using bead‐beating and freeze–thaw cycles. Proteins from lysed cells or vesicles were suspended in RapiGest SF (Waters, Milford, MA, USA) to facilitate protein solubilization and then underwent disulfide reduction with tris(2‐carboxyethyl)phosphine, alkylation with iodoacetamide, and in‐solution protease digestion using either a trypsin/Lys‐C mix or Glu‐C (Promega, Madison, WI, USA). Following RapiGest hydrolysis and desalting, samples were resuspended in a solution containing an internal standard of synthetic peptides (Hi3 *Escherichia coli* standard, Waters), spiked with iRT retention time standard (Biognosys, Boston, MA, USA), and immediately analysed on a Waters ACQUITY M‐class LC coupled to a Thermo QExactive HF HRMS equipped with a nano‐electrospray source. Data‐dependent acquisition was performed on the top 10 ions and data analysis were conducted using the software from the trans‐proteomic pipeline (TPP v.5.1.0) (Nesvizhskii *et al*., 2007).

Label‐free comparison of relative protein abundances was facilitated by normalizing protein spectral counts to the Hi3 internal standard to account for differences in ionization of peptides due to sample matrix affects and then normalizing to the amount of biomass injected onto the LC‐HRMS. To compare protein enrichments within *Prochlorococcus* cell and vesicle fractions, ‘per cell’ or ‘per vesicle’ spectral counts were then normalized on the basis of size, or biovolume, differences between a cell and a vesicle, herein referred to as ‘biovolume‐normalized protein spectral counts’ (Supporting Information Tables S2 and S3). Unless otherwise indicated, proteome comparisons across cells and vesicles were conducted using biovolume‐normalized spectral counts from only trypsin/Lys‐C‐based proteomes (Fig. 2). Relative protein enrichments in *Prochlorococcus* vesicles as compared with cells was computed using log_2_ ratios (Supporting Information Table S4) and displayed in Fig. 2A using only the proteins with spectral counts in the top 25% of those identified in vesicles of strain MIT9312 or MIT9313. Subcellular localization assignments are based on a combination of predictions from the Uniprot database (https://www.uniprot.org), results from the PSORTb algorithm (V3.0.2) (Yu *et al*., 2010), amended with data from TMHMM 2.0 (Krogh *et al*., 2001) and SignalP (V4.1) (Nielsen, 2017), based on the Gram‐negative bacteria model. Proteins with a putative signal peptide but no other significant localization predictions are noted as being ‘unclassified non‐cytoplasmic’. Additional details are available in Supplementary Information.

### Metabolomics

Cell pellets and vesicles originating from triplicate 20 l batch cultures of *Prochlorococcus* MIT9312 or MIT9313 were extracted using a modified Bligh and Dyer technique with cold 1:1 methanol/water (aqueous phase) and cold dichloromethane (organic phase) (Bligh and Dyer, 1959; Boysen *et al*., 2018). Cell pellets were manually disrupted by bead beating during the extraction, as described by Boysen *et al*. (2018); vesicles were extracted without bead beating. Metabolite separations were achieved using reversed‐phase (RP, for aqueous and organic extracts) and hydrophilic interaction liquid chromatography (HILIC, for aqueous extracts only), as detailed in Table S8 and Boysen *et al*., 2018. Data were collected in positive ion mode for RP analyses with a scan range of 90–900 *m/z*. For HILIC analyses, data were obtained in positive and negative mode (using polarity switching) with a scan range of 80–900 *m/z*. For every sample, data were processed in four subgroups, defined by phase, chromatography, and ion mode: RP‐organic‐positive (RPOrgPos), RP‐aqueous‐positive (RPAqPos), HILIC‐aqueous‐positive (HILICAqPos), and HILIC‐aqueous‐negative (HILICAqNeg). Data were collected using Thermo QExactive HF HRMS using same settings as in the study by Boysen *et al*. (2018).

For targeted data, individual metabolite features were integrated using Skyline Daily (MacLean *et al*., 2010) and subjected to an in‐house quality control protocol (Boysen *et al*., 2018). Untargeted data were processed using MS‐DIAL software (Tsugawa *et al*., 2015) using parameters reported in Table S9. MS‐DIAL was used to pick, align, and integrate mass features from raw datasets. Identification of mass features within untargeted data sets was accomplished via dereplication of the mass feature list against several databases, yielding identifications of variable confidence as described in the study by Heal *et al*. (2019). We ranked confidence in mass feature identifications according to existing literature (Sumner *et al*., 2007). All mass features were searched against an Ingalls Lab in‐house database of authenticated standards, LOBSTAHs output, and MassBank (Horai *et al*., 2010). Additional details are available in Supplementary Information.

### Vesicle enzyme activity assays

All nutrient and enzymatic assays were carried out using vesicles collected from 20 l exponentially growing *Prochlorococcus* cultures grown as above. Vesicles were purified using an OptiPrep (Iodixanol) density gradient as described in the study by Biller *et al*. (2014) and washed in clean 1× PBS. ATP was measured using a standard luminescence‐based assay with the BacTiter‐Glo kit (Promega) according to the manufacturer's directions. Vesicle phosphate was measured using the Sigma‐Aldrich Phosphate Assay kit (MilliporeSigma, St. Louis, MO, USA). Protease measurements were made using the Sigma‐Aldrich Protease Fluorescent Detection Kit following the manufacturer's instructions with the following modifications: 10 μl of a 2× incubation buffer was used in each 50 μl reaction, which allowed us to add 20 μl of sample to each reaction and increase the number of vesicles included in the reaction. The reaction was carried out for 18–20 h at 28°C in the dark. Sulfatase activity was measured using the BioVision Sulfatase Activity Assay (BioVision, Milpitas, CA, USA), following manufacturer's instructions.

Phosphatase and lipase activity were measured by following the hydrolysis of fluorescent substrates 4‐methylumbelliferyl phosphate and 4‐methylumbelliferyl oleate (both from Sigma‐Aldrich) following standard methods (Hoppe, 1993). Briefly, ~10^10^ vesicles in 1× PBS were incubated with 100 μM substrate (dissolved in ethylene glycol monomethyl ether) and natural seawater from the Sargasso Sea in a 200 μl reaction within a black‐walled 96‐well plate at room temperature for 16 h. Boiled vesicle samples and substrate in seawater lacking any added vesicles were used as controls.

### Vesicle interaction experiments

All cultures used for the interaction experiments were grown in media based on 0.2 μm filtered natural water from Vineyard Sound, MA except where noted. Vesicles were purified from 20 l cultures of *Prochlorococcus* MIT9312 or MIT9313 grown as above but in Pro99 media (Moore *et al*., 2007), and 10 l cultures of *Alteromonas* strain MIT1002 (Biller *et al*., 2015b) were grown at 24°C in ProMM media (Berube *et al*., 2015) (Pro99 media, as above, plus lactate, pyruvate, glycerol, acetate, and 1× Va vitamin mix; Waterbury and Willey, 1988). The 10 l cultures of *E. coli* strain TB28(*att*HKTB263) (Dinh and Bernhardt, 2011; this strain expresses the Superfolder variant of GFP and targets it to the periplasm) were grown at 30°C in M9 maltose media supplemented with 250 μM isopropyl β‐d‐1‐thiogalactopyranoside and 100 μg m^−1^ ampicillin. Purified vesicles were covalently labelled with an amine‐reactive Alexa Fluor 488 5‐SDP ester dye (Molecular Probes/ThermoScientific).

To examine the ability of vesicles to associate with representative phylogenetically distinct marine microbes, cells of *Prochlorococcus* MIT9312 and MIT9313 (in Pro99 media), *Alteromonas* MIT1002 and *Thalassospira* MIT1004 (Biller *et al*., 2017) (in ProMM media), and *Polaribacter* MED152 (González *et al*., 2008) (in Pro99 media, supplemented with 5 g l peptone and 1 g l yeast extract) were all grown to mid‐exponential growth phase at 24°C. *Pelagibacter* HTCC7211 was grown in defined AMS1 media (Carini *et al*., 2013) at 22°C. Approximately 10^9^–10^10^ vesicles (labelled or unlabelled), or an equivalent volume of the PBS Alexa 488 labelling control, were added to 2 ml of culture at ~10^5^ cells ml^−1^ (final ratio: ~1000:1 vesicles:cells). Cultures with vesicles were incubated for 2 h at the normal growth temperatures and examined on an Influx flow cytometer (Cytopeia/BD, Franklin Lakes, NJ). Cells were excited using a blue 488 nm laser and monitored for chlorophyll (692/40 nm emission) and Alexa 488/GFP (530/40 nm emission) fluorescence. All flow cytometry data were analysed using FlowJo (V10.5). Additional details are available in Supplementary Information.

### Miscellaneous

All data were analysed in R (V3.5.1). Plots were created using ggplot2 (Wickham, 2009).

## Supporting information


**Appendix S1:** Supporting Information

**Supplementary Table S1:** Complete dataset of membrane lipids, pigments, and plastoquinone found within *Prochlorococcus* MIT9312 and MIT9313 cells and vesicles.

**Supplementary Table S2:** Complete proteomic data for *Prochlorococcus* MIT9312 and MIT9313 cells and vesicles, including protein and peptide identification and raw spectral counts.

**Supplementary Table S3:** Relative protein abundances, or biovolume‐normalized spectral counts, for *Prochlorococcus* MIT9312 and MIT9313 cells and vesicles.

**Supplementary Table S4:** Relative enrichment of proteins in *Prochlorococcus* vesicles vs. cells.

**Supplementary Table S5:** Metabolite diversity in *Prochlorococcus* vesicle and cell samples.

**Supplementary Table S6:** Full biovolume normalized peak areas for *Prochlorococcus* vesicles and cells.

**Supplementary Table S7:** Targeted metabolites detected in vesicles and cells from *Prochlorococcus* MIT9312 and MIT9313.

**Supplementary Table S8:** Experimental conditions used to analyse metabolite fractions.

**Supplementary Table S9:** Parameters for MS‐DIAL analysis for each analytical fraction.

## Data Availability

The mass spectrometry proteomics data have been deposited to the ProteomeXchange Consortium via the PRIDE (Perez‐Riverol *et al*., 2019) partner repository with the dataset identifier PXD013602. Metabolite data are available at the NIH Common Fund's National Metabolomics Data Repository (NMDR) website, the Metabolomics Workbench, https://www.metabolomicsworkbench.org (Sud *et al*., 2016), under Study ID # ST001524.
